# Femoral neuropathy following venoarterial-extracorporeal membrane oxygenation therapy: a case report

**DOI:** 10.1186/s12872-020-01675-y

**Published:** 2020-08-27

**Authors:** Albert Youngwoo Jang, Young Jun Oh, Seok In Lee, Oh Kyung Lim, Soon Yong Suh

**Affiliations:** 1 Division of Cardiology, Department of Internal Medicine, Gachon University Gil Medical Center, 1198 Guwol-dong, Namdong-gu, 405-760 Incheon, Republic of Korea; 2 Intensive Care Unit, Department of Nursing, Gachon University Gil Medical Center, Incheon, Republic of Korea; 3 Department of Thoracic Cardiovascular Surgery, Gachon University Gil Medical Center, Incheon, Republic of Korea; 4 Department of Physical & Rehabilitation Medicine, Gachon University Gil Medical Center, Incheon, Republic of Korea

**Keywords:** Femoral neuropathy, Extracorporeal membrane oxygenation, Case report

## Abstract

**Background:**

Although life-threatening complications of extracorporeal membrane oxygenation (ECMO) are well described, non-life threatening complications are less known. Herein, we report a case of femoral neuropathy (FN) due to nerve compression caused by cannula compression and deep vein thrombosis (DVT) after successful ECMO therapy, which seriously undermined one’s quality of life.

**Case presentation:**

A 70-year old male presented to the emergency department for chest pain. The patient had cardiac arrest before percutaneous coronary intervention (PCI) and was inserted with ECMO. Although he was successfully weaned from ECMO 4 days after PCI, he consistently complained swelling, abnormal sensation, and weakness in his right lower extremity, where the cannulas were inserted. Imaging studies showed deep vein thrombosis (DVT) in his right leg, which was further treated with anticoagulants. Symptoms, however, remained after the regression of DVT. Nerve conduction study revealed femoral neuropathy, which may have been caused by ECMO cannula compression and tissue swelling.

**Conclusion:**

The current case proposes that non-life threatening complications of ECMO therapy can seriously affect quality of life. Venous drainage distant from the arterial cannula may prevent such complications.

## Background

Extracorporeal membrane oxygenation (ECMO) is a mechanical circulatory supporting device in patients with critical respiratory, cardiac, or combined failure. Due to great survival benefit, more than 24,000 cases of ECMOs were inserted in adults in the United States in 2019, which is approximately 6 times more compared with 2005 [1]. Life threatening neurologic complications, such as stroke, seizure, or intracranial hemorrhage have been reported [2]; however non-life threatening neurologic complications, such as neuropathy associated with ECMO cannulation are less known. Herein, we report a case of femoral neuropathy (FN) caused by nerve compression and massive swelling due to deep vein thrombosis (DVT) following ECMO cannulation, which seriously undermined the patient’s quality of life.

## Case presentation

A 70-year-old male with a history of hypertension, diabetes mellitus, and atrial fibrillation presented to the emergency department with chest pain lasting for 1 hour. The patient did not have any previous neurologic deficits or surgical, family, or genetic history, although he was a heavy smoker (75 pack-years). His initial blood pressure was 109/62 mmHg with a heart rate of 103 beats per minute. There were no remarkable findings upon physical exam. The electrocardiogram (ECG) showed ST segment elevation in leads II, III, and aVF and reciprocal changes in leads I and aVL, suggestive of ST elevation myocardial infarction. Initial troponin I level (0.021 ng/mL [0–5 ng/mL]) was unremarkable. The patient was started on intravenous unfractionated heparin (UFH).

The patient was immediately moved to the catheterization lab for emergent percutaneous coronary intervention (PCI) of ST elevation myocardial infarction. Vital signs were normal during the femoral artery puncture and insertion of a 6 French (Fr) sheath into the right femoral artery (FA). We punctured at the femoral head level without any mispuncutures during the process. After puncture and before coronary angiography (CAG), however, the patient went into ventricular fibrillation and the blood pressure became unmeasurable. Cardiopulmonary resuscitation (CPR) was immediately initiated with defibrillation every 2 min, although normal rhythm of vital signs were not recovered. As CPR was performed for a total of 30 min, we concurrently inserted a venoarterial ECMO. A 16.5 Fr (external diameter: 5.5 mm) arterial and 21 Fr (external diameter: 7 mm) venous cannula was inserted through the right FA and femoral vein (FV), respectively. CAG revealed an extensive thrombotic occlusion of the mid right coronary artery (RCA) with thrombolysis in myocardial infarction (TIMI) 0 flow distally. A drug eluting stent (Biomime™ 4.0 × 19 mm) was inserted to the lesion and the RCA restored a TIMI 2 flow (pain-to-balloon time: 120 min).

After the PCI, the patient was moved to the intensive care unit for ECMO care. Initial echocardiography showed a left ventricular ejection fraction (LVEF) of 15% and an extensive regional wall motion abnormality of the RCA territory. However, there was blood pressure drop with concurrent massive nasal and gastrointestinal bleeding. Hemoglobin level became 5.9 g/dL, which was approximately 8 g/dL decrease compared with initial levels. Blood pressure was recovered to normal after massive transfusion; however, we lowered the target activated partial thromboplastin time to approximately 40 s to prevent additional bleeding. There were no cannulation site complications such as hematoma or signs of infection during ECMO care, although diffuse swelling developed in the right lower extremity (LE) the next few days. Pneumatic compression devices were applied to both LEs to prevent DVT. ECMO therapy was maintained for 4 days, while vital signs slowly recovered. As the LVEF was restored to 46% on the 4th day, the ECMO was weaned and removed. The ECMO cannulas were removed by manual compression. Despite the risk of stent thrombosis, dual-antiplatelet therapy was stopped after ECMO removal (day 4) in concern of additional bleeding.

**Fig. 1:**
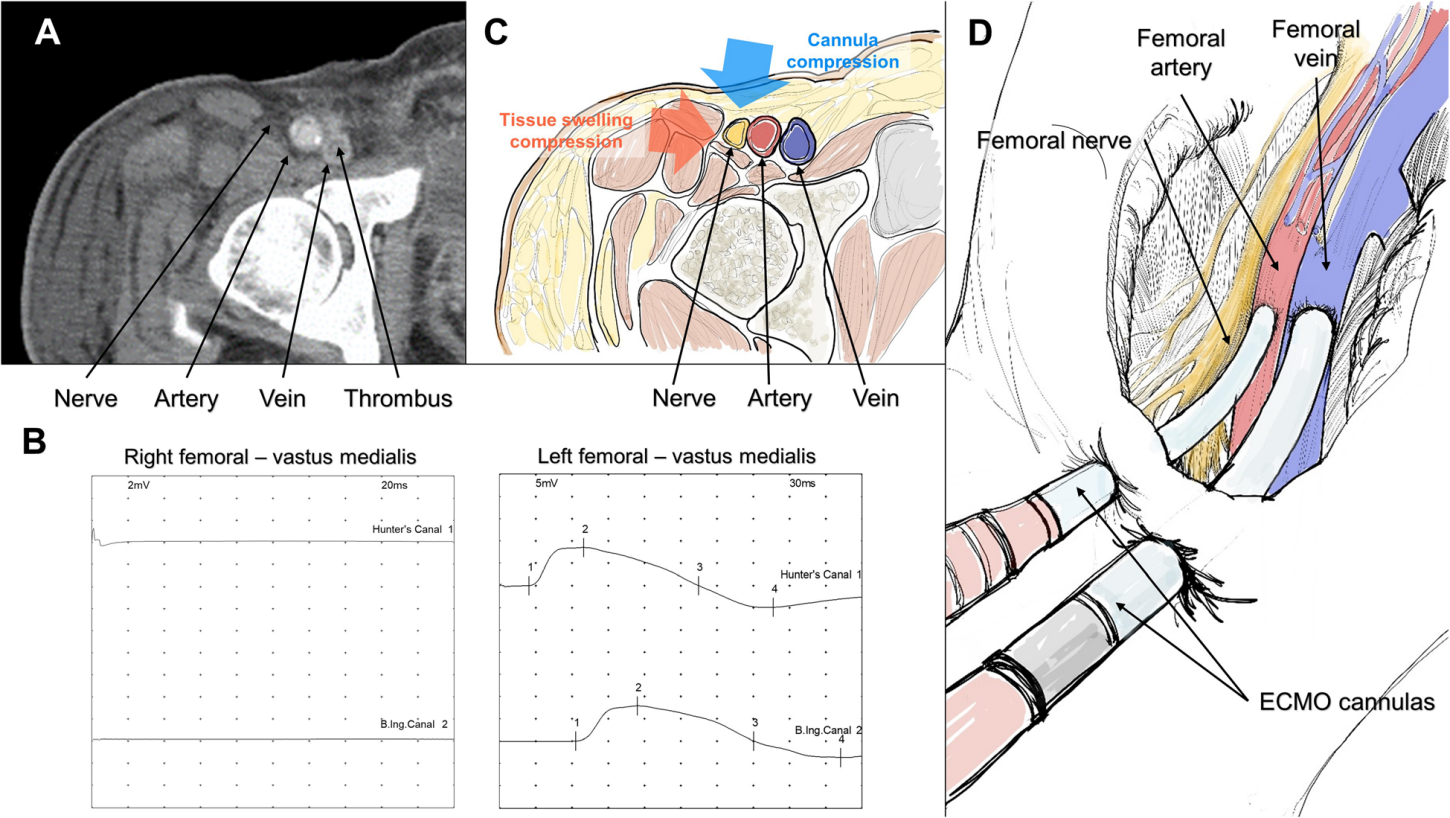
Computerized tomography image, schematic diagram, and nerve conduction study results. A computerized tomography image of the femoral artery and vein area, 2 weeks after extracorporeal membrane oxygenation (ECMO) cannula removal showing severe right leg swelling and intense residual thrombus within the femoral vein (**a**). Impaired conduction in the right femoral nerve conduction study is shown, compared with the left femoral nerve (**b**). A cross-sectional schematic image of the femoral area showing 1) external compression of the sheath within the vessels; and 2) severe generalized swelling of the right lower extremity causing compression to the femoral nerve (**c**). An illustration of arterial and venous sheaths of the ECMO inserted into the femoral and artery and vein, respectively, where large sheaths may cause external compression of the femoral nerve (**d**)

On the 7th day of admission, the patient recovered orientation. However, leg edema did not improve despite ECMO removal. He also started complaining impaired function, pain, and hypesthesia of the LE. Compartment syndrome was initially suspected. We clinically ruled out compartment syndrome [3], since the patient was negative for Homan’s sign and had good distal pulsation with warm circulation. The patient had burning sensation and Grade 3 weakness in hip and knee flexion motions with no deep tendon reflex. Neurologic findings were not dermatome specific. LE Doppler sonography and computed tomography (CT) showed DVT extending from the right external iliac vein to the calf vein without any evidence of puncture site hematoma or intramural bleeding (Fig. 1a). The patient was started on rivaroxaban (15 mg twice daily) for DVT (day 8). The swelling of the right LE gradually improved over the next 30 days, although his pain and weakness were not alleviated. As the patient was suspected to have post-thrombotic syndrome (PTS), which is pain and abnormal sensation after the onset of DVT, further studies such as the nerve conduction study (NCS) or electromyography were not considered until later on. The patient was discharged to a rehabilitation hospital on the 35th day.

During outpatient department follow up, the patient consistently had pain, abnormal sensation, and weakness in the right LE, which resulted in insomnia and depression. Although he was given thioctic acid (600 mg qd) and pregabalin (150 mg bid) during outpatient department follow up, his pain remained. Femoral and pulmonary arterial CT angiography was performed 100 days post-ECMO insertion showed no thrombus in the right LE or pulmonary arteries. However, the NCS revealed no sensory nerve action potential in the right peroneal nerve and tibial nerve, suggestive of impaired sensation. Additionally, the compound muscle action potential was not observed in the right femoral (Fig. 1b), peroneal and tibial nerve, indicating motor nerve palsy. These results collectively suggested FN. The patient is continuing rehabilitation exercises and slowly recovering from the weakness, although the tingling sensation remains to a lesser degree. The patient shares his regrets on his previous smoking habits which caused his myocardial infarction and eventually FN. An informed consent for publishing data was obtained from the patient. A timeline of events is summarized in Table 1.

**Table 1:** A timeline of events: symptoms, diagnosis, and treatment

Event	Timeline
Chest pain and ED presentation	Day 1
Cardiac arrest	Day 1
CPR and VA ECMO insertion (right FA and FV)	Day 1
Intravenous UFH infusion started	Day 1
PCI to mid RCA	Day 1
Pneumatic compression device started	Day 1
Swelling of right LE	Day 2
ECMO removal	Day 4
Discontinuation of UFH and DAPT	Day 4
Recovery of consciousness and orientation	Day 7
Patient starts complaining impaired function, pain, and hypesthesia of right LE	Day 7
DVT diagnosis by Doppler sonography and CT	Day 8
Initiation of antiplatelet therapy	Day 25
Regression of swelling of right LE	Day 35
PTS suspected: patient discharged with pain and weakness in right LE remaining	Day 35
Nerve conduction study reveals femoral neuropathy	Day 100
Patient continues rehabilitation and symptoms improve	Day 200

*ED* emergency department, *CPR* cardiopulmonary resuscitation, *VA* venoarterial, *ECMO* extracorporeal membrane oxygenation, *FA* femoral artery, *FV* femoral vein, *UFH* unfractionated heparin, *PCI* percutaneous coronary intervention, *RCA* right coronary artery, *LE* lower extremity, *DVT* deep vein thrombosis, *CT* computerized tomography, *PTS* post-thrombotic syndrome

## Discussions and conclusions

As the clinical outcomes of ECMO therapy is improving, attention to the complications that determine the post-ECMO quality of life are emerging. Neuropathy caused by limb ischemia [4] or compartment syndrome [5] following ECMO therapy have been observed. However, FN caused by DVT and/or cannula-related nerve compression after ECMO therapy have not been reported.

FN is a rare complication most associated with intra-abdominal or hip surgery. Prolonged pneumatic compression by the tourniquet also causes FN. The mainly suggested mechanism is stretch or prolonged compression of the nerve resulting in microvascular congestion, impaired tissue perfusion, and axonal degeneration [6, 7], leading to transient or permanent pain, paresthesia, or loss of function. FN can be confirmed by an NCS or electromyography. FN can be differentiated from critical illness neuropathy (CIP), because CIP is usually manifested with symmetry involving all extremities. Physical therapy is the mainstay of FN treatment unless the cause of compression can be removed, such as a tumor. Anatomically, the femoral nerve is located within the femoral triangle, which contains the femoral nerve, FA, and FV. The femoral nerve, FA and the FV is located from lateral to medial, where the lateral border is the sartorius and medial border is the abductor longus muscle.

FN in the current case is suspected to be caused by nerve compression due to two bulky inserted cannulas and diffuse swelling of surrounding tissues caused by PTS. Although ipsilateral arterial and venous ECMO cannulation (cannulation of both arterial and venous sheaths in the same leg) is frequently done in the real-world practice [2, 8], the related complications are not known. Large diameter cannulas inserted in the ipsilateral side may increase the risk of compression of the adjacent structures such as the nerve (Fig. 1a, c, and d). Additionally, PTS caused by DVT may have aggravated the compression of the nerve. PTS is an often-overlooked complication of DVT, which is caused by the injury of endothelium and low venous flow. It leads to the distension of collateral vessels and general swelling of the tissue, which may result in pain and cramping [3]. DVT is also frequently observed after ECMO decannulation [9]. Routine evaluation of DVT may be necessary to prevent further complications [9]. We suspect that the femoral nerve already compressed by both large cannulas may have been additionally pressured by the general swelling of the tissues (Fig. 1c-d). Immediately after the removal of cannulas, PTS was initially suspected. However, as the symptoms were consistent even after the improvement of DVT, we concluded that the symptoms may have been caused by FN.

The placement of the venous catheter in a distant site may possibly reduce the compression of the nerve. As both artery and venous cannulas inserted in the same area is thought to cause compression of adjacent structures, locating the drainage cannula in the superior vena cava through the internal jugular vein (IJV) or the contralateral FV may reduce such disadvantages. In fact, some studies assert that the venous drainage catheter should be placed in the IJV for better circulation of oxygenated blood [10]. Although initial jugular access for ECMO therapy may be cumbersome in emergent situations, placing the jugular cannula in the contralateral side may be a feasible option. Early rehabilitation in patients with damaged nerves has been shown to improve outcomes and shorten the duration of recovery of FN. However, the rehabilitation in our patient was delayed to prevent DVT progressing to acute pulmonary thromboembolism. Also, the medical staff in the rehabilitation hospital were reluctant of conducting aggressive rehabilitation in a patient with a history of cardiac arrest. We suspect that such circumstances may have resulted in the slow recovery of FN.

The importance of complications that determine the quality of life of the patient after ECMO therapy is emerging, as the outcomes of ECMO therapy are improving [11]. The current case proposes that FN can seriously undermine one’s quality of life even after successfully weaning from ECMO. Considering venous drainage in the vessel distant from the arterial cannula or early rehabilitation may be critical in preventing such a detrimental complication.

## Data Availability

The datasets generated and/or analyzed during the current study are not publicly available due to privacy reasons.

## Abbreviations

ECMO: Extracorporeal membrane oxygenation
FN: Femoral neuropathy
DVT: Deep vein thrombosis
ECG: Electrocardiogram
Fr: French
CAG: Coronary angiography
RCA: Right coronary artery
LE: Lower extremity
CT: Computed tomography
PTS: Post-thrombotic syndrome
NCS: Nerve conduction study
IJV: Internal jugular vein
